# ITGAL expression in non-small-cell lung cancer tissue and its association with immune infiltrates

**DOI:** 10.3389/fimmu.2024.1382231

**Published:** 2024-04-04

**Authors:** Ruihao Zhang, Guangsheng Zhu, Zaishan Li, Zhenzhen Meng, Hua Huang, Chen Ding, Yanan Wang, Chen Chen, Yongwen Li, Hongyu Liu, Jun Chen

**Affiliations:** ^1^ Department of Lung Cancer Surgery, Tianjin Medical University General Hospital, Tianjin, China; ^2^ Department of Cardiothoracic Surgery, Linyi People’s Hospital, Linyi, China; ^3^ Department of Anesthesiology, Linyi People’s Hospital, Linyi, China; ^4^ Tianjin Key Laboratory of Lung Cancer Metastasis and Tumor Microenvironment, Tianjin Lung Cancer Institute, Tianjin Medical University General Hospital, Tianjin, China

**Keywords:** ITGAL, immune microenvironment, NSCLC, biomarker, immune cell

## Abstract

**Background:**

Integrin subunit alpha L (ITGAL) encodes an integrin component of LFA-1 and is a membrane receptor molecule widely expressed on leukocytes. It plays a key role in the interaction between white blood cells and other cells. There was a significant correlation between the expression of ITGAL and the tumor microenvironment in a number of cancers. However, experimental studies targeting ITGAL and immune cell infiltration in non-small-cell lung cancer (NSCLC) and the response to immune checkpoint inhibitor therapy are lacking.

**Methods:**

Data were obtained from The Cancer Genome Atlas (TCGA), Gene Expression Omnibus (GEO), and Clinical Proteomic Tumor Analysis Consortium (CPTAC) databases to explore the relationship between ITGAL expression and prognosis, as well as the immune cell infiltration in patients with NSCLC. In addition, immunohistochemical staining for ITGAL and multiplex immunofluorescence (mIF) staining for ITGAL, CD20, CD68, CD4, and CD8 from tissue microarrays containing 118 tumor tissues and paired paracancerous tissues from patients with NSCLC were performed. The correlation between ITGAL expression and clinical factors, as well as the immunophenotypes of tumor-infiltrating immune cells, were also analyzed.

**Results:**

In NSCLC tumor tissues, ITGAL was downregulated compared with matched paracancerous tissues, and low ITGAL expression was associated with a poor prognosis of NSCLC patients. Subsequently, immunohistochemistry results for tissue microarray showed that ITGAL expression was mainly elevated in tumor stroma and areas with highly infiltrated immune cells. ITGAL expression was higher in paracancerous tissues than tumor tissues. Furthermore, mIF results indicated that the patients with ITGAL-high expression tend had significantly higher CD8+ T cells, CD68+ macrophages, CD4+ T cells, and CD20+ B cells infiltration in their tumor tissues. Immunophenotypes were classified into three categories, that is deserted, excluded, and inflamed types, according to each kind of immune cell distribution in or around the cancer cell nest. MIF results showed that ITGAL expression level was correlated with the immunophenotypes. Furthermore, ITGAL expression was associated with the prognosis of NSCLC in patients with immune checkpoint inhibitor therapy and the patients with high ITGAL expression tends have better outcomes.

**Conclusions:**

ITGAL may be used as a biomarker for assessing the immune microenvironment in patients with NSCLC.

## Introduction

Approximately 2.1 million new cases of lung cancer are diagnosed each year, and 1.8 million deaths are caused by this disease (1). Overall, lung cancer consists of two major groups: small-cell lung cancer (SCLC) and non-small-cell lung cancer (NSCLC), which account for about 15% and 85% of lung cancer cases, respectively. Of these, lung adenocarcinoma (LUAD) is the predominant subtype of NSCLC cases (2, 3). In spite of significant advances in diagnosis and treatment technology, NSCLC still has a very low overall cure rate and survival rate, especially when it is metastatic (4). In current cancer treatment, immunotherapy has been shown to be effective in treating a wide range of cancer types, including NSCLC (5, 6). However, not all NSCLC patients benefit from immunotherapy, which may be related to the tumor’s immune microenvironment. In order to develop new immunotherapy targets for NSCLC, it is imperative to identify specific immune-related molecules.

ITGAL, also known as integrin αLβ2 (CD11a/CD18, CD11a), encodes an integrin component of LFA-1 and is a membrane receptor molecule widely expressed on leukocytes (7). ITGAL is an important molecule in the interaction between leukocytes and other cells (e.g., endothelial cells), and its main function is to participate in cell adhesion and migration, which are important for cellular immune responses, inflammatory responses, etc. (8–10). Recent studies have discovered that ITGAL is enriched in the tumor microenvironment, drawing much interest in oncology (11–13). ITGAL was reported to promote leukocyte migration and adhesion through an increase in the expression of Cx3cr1 and Ccl5, which in turn control leukocyte migration and mitogen production (14). Upregulation of ITGAL expression was strongly associated with immunomodulators, chemokines, and levels of CD8+ T cells, CD4+ T cells, B cells, monocytes, neutrophils, macrophages, T cells, natural killer (NK) cells, and myeloid dendritic cell infiltration in gastric adenocarcinomas (STAD). Further, some studies indicate that ITGAL might play a key role in cancer growth and transformation, making it a potentially useful target in the treatment of a wide variety of cancers (15–17). The main effect of resveratrol on NSCLC treatment may be related to immune signaling pathways, according to a mechanistic study of resveratrol in NSCLC (18). In NSCLC, the activated T cell (CD4 memory) pathway is mainly responsible for regulating the immune microenvironment, and ITGAL expression is significantly correlated with the immune microenvironment, according to some studies (19). In spite of this, the possible mechanisms of ITGAL on tumor development and immune interaction with NSCLC are still unknown, and experimental studies addressing the correlation between ITGAL and immune cell infiltration are limited.

We used immunohistochemical staining to diagnose ITGAL expression in 118 samples of NSCLC tissue and paraneoplastic tissue. Multiplex immunofluorescence staining (mIF) was also performed on the tissue microarrays to explore the relationship between ITGAL expression and infiltration of several immune cells, as well as the possible immunomodulatory role of ITGAL in NSCLC. We aim to uncover the critical role ITGAL plays in NSCLC, the possible connection between ITGAL and tumor-infiltrating immune cells, and the mechanism by which ITGAL may affect this process.

## Materials and methods

### NSCLC tissue samples and NSCLC tissue microchip

The Department of Lung Cancer Surgery of Tianjin Medical University General Hospital (TMU) collected lung cancer tissue and paraneoplastic lung tissue from five patients with NSCLC. Tissue microchips containing 118 pairs of primary lung cancer tissues and corresponding adjacent cancerous tissues were purchased from Saville Biotech (Wuhan, China). Each patient provided informed consent, and the study was approved by the Tianjin Medical University Ethics Committee.

### Immunohistochemistry

The sections were first deparaffinized and hydrated, and antigen retrieval was achieved using a sodium citrate buffer before incubation with anti-human CD11a/integrin alpha L polyclonal antibody (dilution, 1:150) [(15574-1-AP), Proteintech] overnight at 4°C. TBST was added three times to the slide, followed by the addition of rabbit anti-igG (Proteintech). The slide was incubated for 1 hour at room temperature after being washed three times in TBST. After washing the slides with phosphate-buffered saline (PBS), diaminobenzidine was used to expose them, and then hematoxylin was used to counterstain them. The immunohistochemistry (IHC) results were categorized as negative (-) and positive (+) according to the degree of staining, and each tissue was quantified according to the degree and extent of immunohistochemical staining using a 12-point counting method (0, 1, 2, and 3 according to the degree of staining, multiplied by the extent of staining, 1, 2, 3, and 4). In a blind review of the staining results, two pathologists without prior knowledge of clinicopathological data evaluated the results independently.

### Multiplex immunofluorescence

Multiplex immunofluorescence (mIF) staining and primary evaluation were performed by Yucebio Technology (Shenzhen, China). Xylene was used to dewaxe the 5-millimeter slides produced from formalin-fixed, paraffin-embedded (FFPE) blocks. We then rehydrated the slides with decreasing concentrations of ethanol, then fixed them for 10 minutes with 10% neutral buffered formalin. Next, the slides were stained with markers of CD20 (E7B7T) XP^®^ rabbit monoclonal antibody (mAb) (48750S, Cell Signaling Technology), recombinant anti-CD4 antibody [EPR6855, (ab133616), Abcam], anti-CD8 monoclonal antibody [4B11, (MA1-80231), Thermo Fisher Scientific], CD11a/integrin alpha L polyclonal antibody (15574-1-AP, Proteintech), anti-CD68 [KP1, (ZM-0060), ZSGB-Bio], anti-pan cytokeratin antibody [KRT/1877R, (ab234297), Abcam], followed by incubation with blocking proteins for 10 minutes. A secondary polymer antibody conjugated to horseradish peroxidase (HRP) was applied after blocking, followed by tyramide signal amplification (TSA). DAPI staining of the slides was carried out for 10 minutes, followed by imaging using Vectrapolaris (Akoya Biosciences).

### Data source and bioinformation analysis

TCGA (https://portal.gdc.cancer.gov/ (accessed on 20 Nov 2023) and GEO (https://www.ncbi.nlm.nih.gov/geo/ (accessed on 20 Nov 2023) databases were used to obtain seven datasets: TCGA-LUAD contained 510 LUAD samples; TCGA-LUSC contained 498 LUSC samples; GSE116959 contained 57 tumor samples and 11 normal samples; GSE68571 contained 86 tumor samples and 10 normal samples; GSE30219 contained 59 tumor samples and 14 normal samples; GSE33532 contained 16 tumor samples and 20 normal samples and GSE93157 contained 35 non-small cell lung cancer samples, 5 head and necks quamous cellcarcinoma, and 25 melanoma samples. The mRNA expression and clinical correlation of ITGAL in LUAD and LUSC were analyzed using UALCAN (http://ualcan.path.uab.edu/ (accessed on 20 Nov 2023) website and TCGA database. ITGAL expression and its correlation with immune cells, immune scores, and immune infiltration were analyzed using the Sangerbox tool. We analyzed overall survival (OS) and progression-free survival (PFS) using Kaplan-Meier plotting websites.

### Statistical analysis

GraphPad Prism 8 (GraphPad Software, La Jolla, CA) was used for statistical analyses. Data from the three groups of repeated experiments were presented as mean ± standard deviation and independent samples, t-tests were used to analyze the quantitative data. Tables used chi-square tests to detect differences between groups, and box plots used the unpaired Wilcoxon method to test for differences between groups. Survival analyses We calculated the optimal cut-off value for ITGAL using the R package maxstat and assessed prognostic differences between samples using the logrank test. P < 0.05 was considered a statistically significant difference.

## Results

### ITGAL expression was downregulated in primary NSCLC tissue

**Figure 1:**
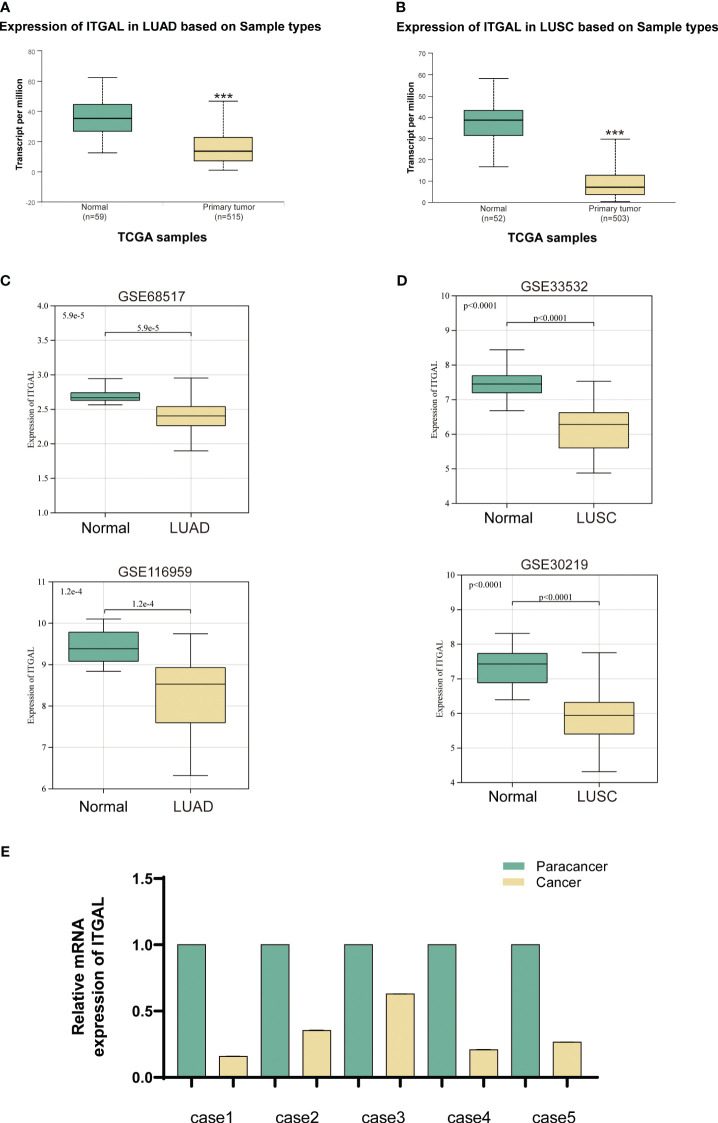
ITGAL expression was downregulated in primary NSCLC tissue. **(A, B)** Expression of ITGAL mRNA in LUAD and LUSC tissues in the TCGA database. **(C)** Verification of ITGAL expression in LUAD tumor tissues in the GEO database (GSE116959 and GSE68517). **(D)** Verification of ITGAL expression in LUSC tumor tissues in the GEO database (GSE33532 and GSE30219). **(E)** Detection of ITGAL mRNA levels in tissue samples from five patients with NSCLC. ***p<0.001.

In order to determine whether human ITGAL plays an oncogenic role in NSCLC, the TCGA and GEO databases were used to examine expression levels of ITGAL in LUAD and LUSC. A significant difference was observed between tumor and paraneoplastic lung samples for ITGAL mRNA expression in TCGA-LUAD and TCGA-LUSC(P<0.001) (**Figures 1A, B**). For further validation, we detected the expression of ITGAL in LUAD by GEO databases, GSE68517 and GSE116959, and in LUSC by databases GSE33532 and GSE30219. A significant reduction in ITGAL protein levels was observed in LUAD and LUSC compared to normal tissues (**Figures 1C, D**). In a subsequent study, we examined the expression of ITGAL mRNA in tumor and peritumor lung tissue samples from five patients with NSCLC. Tissues from tumors expressed less ITGAL than healthy tissues (**Figure 1E**).

### Expression of ITGAL protein and correlation with clinical factors in lung cancer tissue

**Figure 2:**
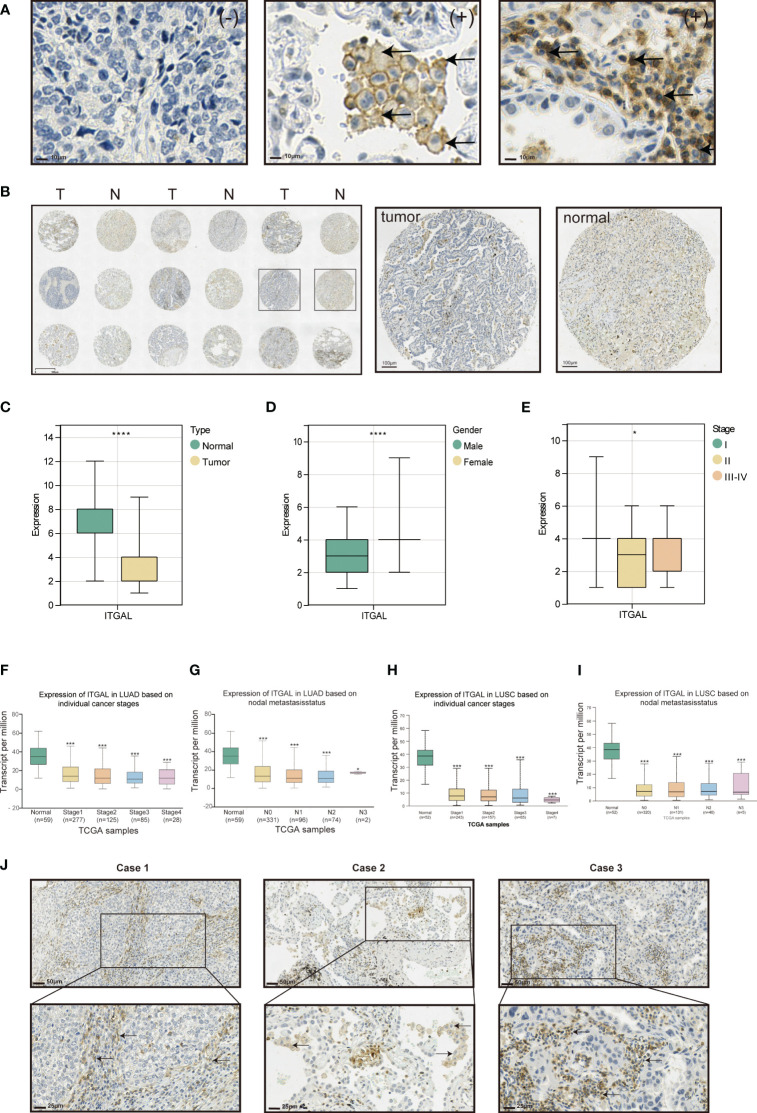
Expression of ITGAL protein and correlation with clinical factors in lung cancer tissue. **(A)** Immunohistochemical staining of ITGAL in lung cancer tissue microarrays. **(B)** Expression of ITGAL in tumor tissues and corresponding normal tissues in lung cancer tissue microarrays. **(C)** Immunohistochemical staining scores of ITGAL in lung cancer tissue microarrays stratified by **(D)** histology type, **(E)** gender, and **(F)** stage. **(F–I)** ITGAL expression levels in patients with LUAD and LUSC stratified by tumor stage and lymph node metastasis status in the TCGA database. **(J)** Expression of ITGAL in lung cancer tissue microarray tumor tissues. *p<0.05, ***p<0.001, ****p<0.0001.

Tissue microarrays containing tissue from 118 patients with primary NSCLC and paired paracancerous tissues were used for ITGAL IHC staining and scoring. As shown in the table below, the tissue microarrays contained characteristics about patients with lung cancer (**Table 1**). The IHC results showed that ITGAL was mainly distributed in the plasma membrane and the cell membrane, with a patchy or nested distribution. In accordance with the median score of ITGAL, patients were divided into two groups: those with high ITGAL expression and those with low ITGAL expression (**Figure 2A**). The following table presents the results of the ITGAL analysis stratified by the median immunohistochemical score into high and low expression for each clinical factor (**Table 2**). By analyzing the IHC results of each patient, we found that paraneoplastic tissues had significantly higher ITGAL expression compared to matched tumor tissues (**Figures 2B, C**). The expression of ITGAL was higher in tumor tissues from female patients than male patients, and in tumor tissues from early-stage patients than advanced-stage patients (**Figures 2D, E**). In order to further validate our findings, we examined ITGAL expression at various stages of NSCLC tumor progression and lymph node metastases using the TCGA database. The ITGAL expression of LUAD and LUSC at stratified clinical stages were lower than that of paracancer normal tissues (**Figures 2F, H**). Meantime, the ITGAL expression of LUAD and LUSC at stratified N stages were lower than that of paracancer normal tissues (**Figures 2G, I**).

**Table 1:** Clinical characteristics of patients with lung cancer tissue microarrays.

Characteristics	Tumor (N=118)
Age	
<=61	59 (50%)
>61	59 (50%)
Gender	
Female	65 (55.08%)
Male	53 (44.91%)
Pathological diagnosis	
Other	10 (8.47%)
Adenocarcinoma	90 (76.27%)
Squamous carcinoma	18 (15.25%)
Stage	
I-II	91 (77.12%)
III-IV	27 (22.88%)

**Table 2:** Immunohistochemical results of ITGAL immunohistochemistry of lung cancer tissue microarrays and their relationship with clinic pathophysiological factors.

Characteristics	ITAGL		Total (N=118)
	low (N=82)	high (N=36)	
Pathological diagnosis			
Other	6 (60%)	4 (40%)	10
Adenocarcinoma	65 (71.43%)	26 (28.57%)	91
Squamous carcinoma	10 (58.82%)	7 (41.18%)	17
Age			
<=61	41 (69.49%)	18 (30.51%)	59
>61	40 (67.80%)	19 (32.20%)	59
Gender			
Female	44 (67.70%)	21 (32.30%)	65
Male	37 (69.81%)	16 (30.19%)	53
Stage			
I-II	62 (68.13%)	29 (31.87%)	91
III-IV	19 (70.37%)	8 (29.63%)	27

Moreover, ITGAL was prominently highly expressed in the stroma area of the tumor tissues and on the membrane and cytoplasm of macrophages and lymphocytes aggregated in these areas (**Figure 2J**).

### NSCLC patients with high expression of ITGAL had a better prognosis

**Figure 3:**
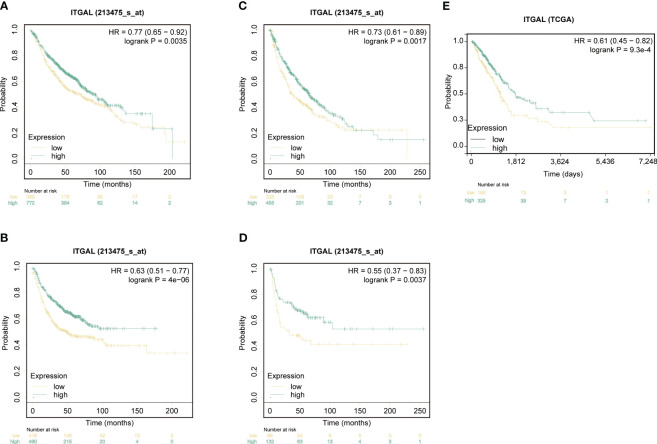
ITGAL expression correlated with prognosis in patients with non-small-cell lung cancer. **(A)** Kaplan-Meier survival curves for overall survival (OS) based on ITGAL expression levels in LUAD. **(B)** Kaplan-Meier survival curves for progression-free survival (PFS) based on ITGAL expression levels in LUAD. **(C)** Kaplan-Meier survival curves for OS based on ITGAL expression levels in LUSC. **(D)** Kaplan-Meier survival curves for PFS based on ITGAL expression levels in LUSC. **(E)** The effect of ITGAL on overall survival in LUAD was analyzed in the TCGA database.

KM survival curves were used to analyze the NSCLC prognosis based on ITGAL expression. In lung adenocarcinomas, patients with higher levels of ITGAL expression had a higher OS rate (HR=0.74, P=6.7e^-03^) and PFS rate (HR=0.73, P=3.3e^-07^). The prognosis was better when ITGAL expression was high (**Figures 3A, B**). Similarly, in lung squamous cell carcinoma, patients with higher expression of the ITGAL gene had longer OS time (HR=0.73, P=1.7e^-03^) and PFS time (HR=0.55, P=3.7e^-03^) (**Figures 3C, D**). A similar result was found in the TCGA database when analyzing OS among patients with LUAD. Those with high expression of ITGAL had a better prognosis (HR=0.61, P=9.3e^-04^) (**Figure 3E**).

### ITGAL expression was correlated with immune infiltrate patterns of NSCLC tumor tissue

**Figure 4:**
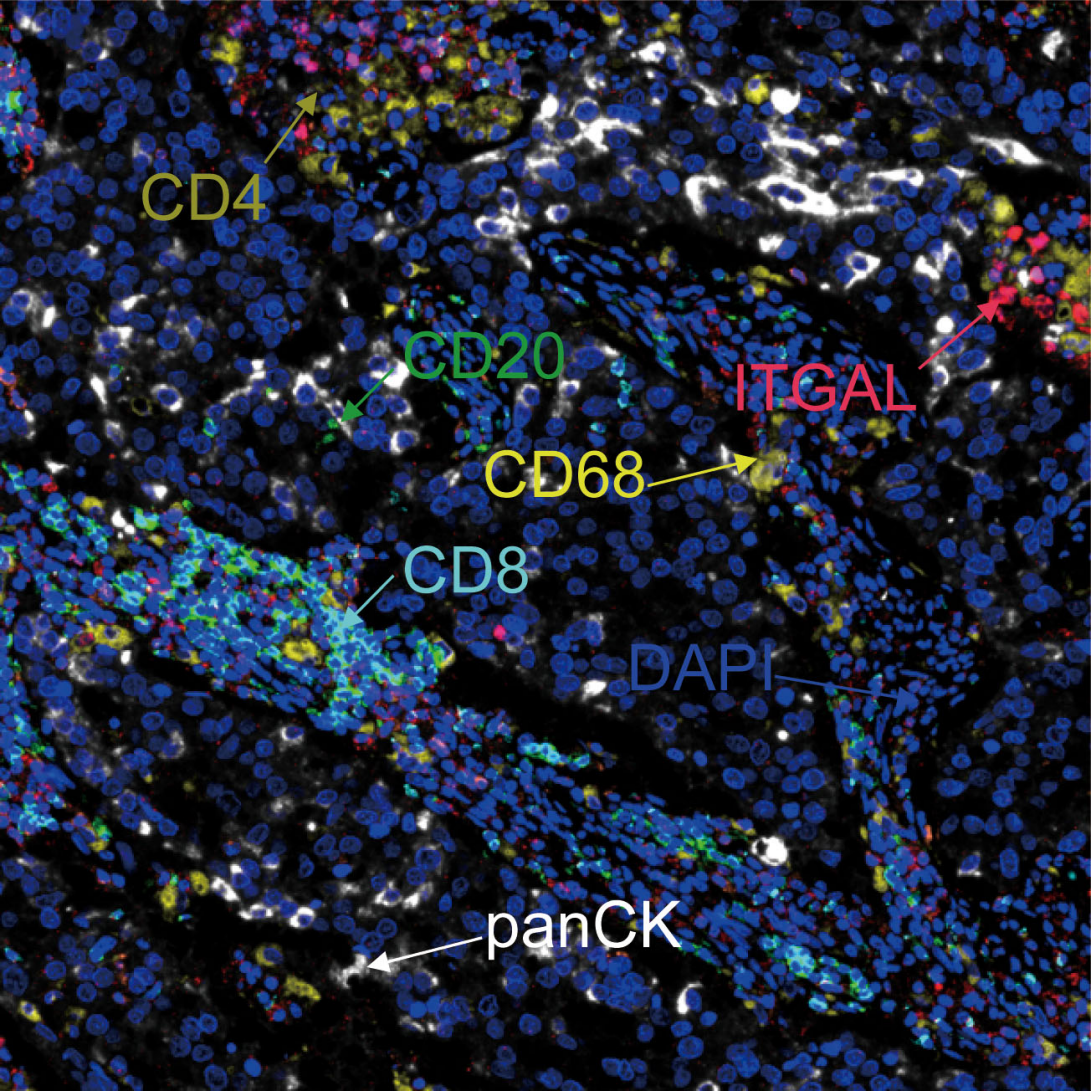
Representative diagram of mIF staining.

**Figure 5:**
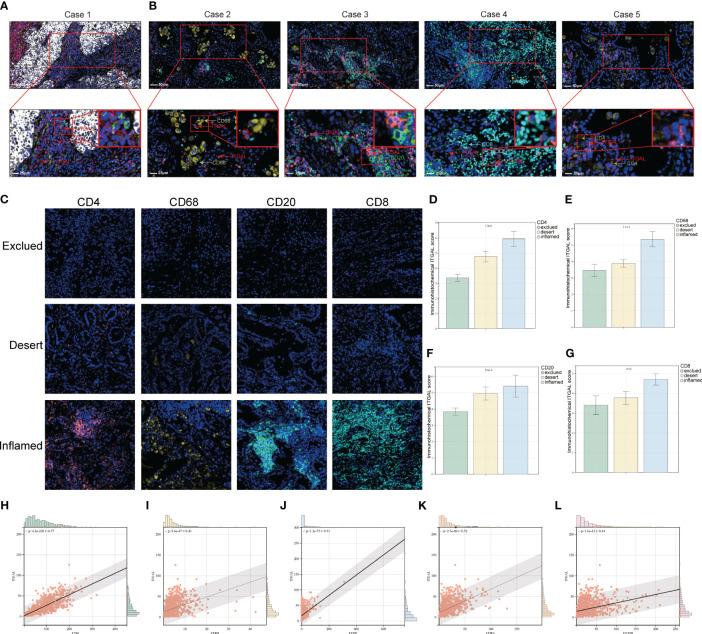
Relationship between ITGAL expression and pattern of immune infiltration in NSCLC tumor tissue. **(A, B)** Multicolor immunofluorescence assay to detect the location of ITGAL expression and immune cells in lung cancer tissue microarray tumor tissues. **(C)** Patients with lung cancer were categorized into inflamed, excluded, and deserted immunophenotypes according to the degree of infiltration of CD20, CD8, CD4, and CD68-positive cells. **(D–G)** Comparison of ITGAL-positive cell infiltration in patients with different immunophenotypes. **(H–L)** Relationship between different immune cell surface markers and ITGAL expression in the TCGA database.

With the help of mIF, we examined the expression of ITGAL in tissue microarrays and the distribution of tumor-infiltrating immune cells. **Table 3** lists the labeled antibodies used and the cells and/or cellular components that were recognized. **Figure 4** shows a representative diagram of mIF staining. In accordance with the immunohistochemical analysis, high levels of ITGAL were detected in tumor stroma (**Figure 5A**). At the same time, we found that areas of high ITGAL expression in NSCLC tumor tissues had different immune cell aggregates or high expression of immune cell markers (**Figure 5B**). Furthermore, we grouped the patients into ITGAL-high and ITGAL-low groups based on the median expression of ITGAL. As shown in **Table 4**, patients with ITGAL-high expression had significantly higher infiltration of CD8+ T cells, CD68+ macrophages, CD4+ T cells, and CD20+ B cells in their tumor tissues, while patients with ITGAL-low expression had significantly lower infiltration of CD8+ T cells, CD68+ macrophages, CD4+ T cells, and CD20+ B cells in their tumor tissues (**Table 4**).

**Table 3:** Multicolor immunofluorescence labeling of antibodies and the cells they recognize.

Marker	Antibodies	Color	Cells/Components
1	CD20	Opal-520	B-cell
2	CD4	Opal-620	Helper T-cell
3	CD8	Opal-480	Cytotoxic T-cell
4	CD68	Opal-570	Macrophage
5	PanCK	Opal-780	Tumor epithelial cell
6	DAPI	DAPI	Nucleus

**Table 4:** Immunohistochemical results of ITGAL immunohistochemistry of lung cancer tissue microarrays and their relationship with staining results of various indicators in tumor tissues.

Characteristics	ITAGL		pvalue
	high (N=36)	low (N=82)	
CD8			0.030
Mean ± SD	10.42 ± 9.61	7.60 ± 9.38	
Median [min-max]	8.46 [0.15,47.86]	3.88 [0.09,45.30]	
CD20			<0.001
Mean ± SD	2.61 ± 4.57	1.36 ± 3.08	
Median [min-max]	0.90 [0.0e+0,21.75]	0.15 [0.0e+0,12.44]	
CD68			<0.001
Mean ± SD	6.13 ± 5.08	3.15 ± 3.05	
Median [min-max]	4.56 [0.08,19.13]	2.35 [0.11,14.86]	
CD4			<0.001
Mean ± SD	4.61 ± 5.05	1.31 ± 2.66	
Median [min-max]	2.88 [0.0e+0,21.82]	0.39 [0.0e+0,19.36]	

Furthermore, immunophenotypes were classified into three categories according to each kind of immune cell distribution in or around the cancer cell nest: deserted, excluded, and inflamed types. ITGAL expression levels for individual immune cells were compared for each immunophenotype (**Figure 5C**). ITGAL expression level is correlated with immune cell immunophenotypes, according to the results. For example, the CD8, CD20, CD68, and CD4-positive exclusion group had the lowest degree of ITGAL-expressing cell infiltration in lung cancer tissues, whereas the inflamed group had the highest degree of ITGAL-expressing cell infiltration in lung cancer tissues. It is therefore possible that ITGAL expression is related to the infiltration of CD4+ and CD8+ T cells, macrophages, and B cells in the lung (**Figures 5D-G**). We examined the correlation of different immune cell surface markers with ITGAL expression using the TCGA database, and similarly, we found that the expression of CD4, CD68, CD20, CD8A, and GZMB was positively correlated with ITGAL expression (**Figures 5H-L**).

### ITGAL expression was associated with the prognosis of NSCLC in patients on immune checkpoint inhibitor therapy

**Figure 6:**
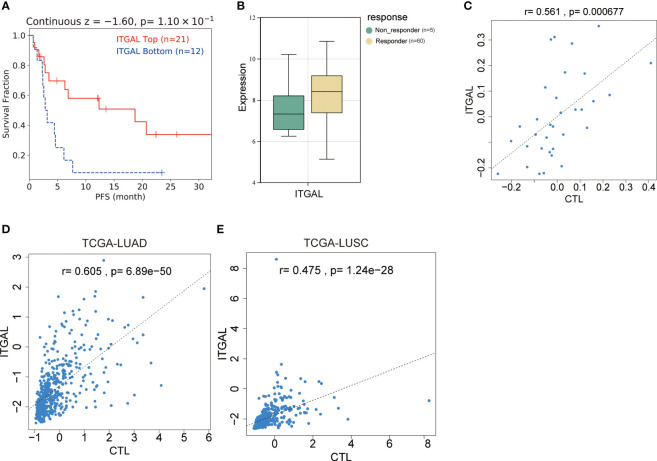
Relationship between ITGAL expression and prognosis of NSCLC in patients treated with immune checkpoint inhibitors. **(A)** Survival curve analysis of patients with lung adenocarcinoma receiving immunotherapy. **(B)** ITGAL expression in patients with lung adenocarcinoma who were responders and non-responders to immunotherapy. **(C)** Correlation analysis of ITGAL gene expression with TIDE score in patients with lung adenocarcinoma receiving immunotherapy. **(D)** Correlation analysis of ITGAL gene expression with the TIDE score in LUAD from the TCGA database. **(E)** Correlation analysis of ITGAL gene expression with the TIDE score in LUSC from the TCGA database.

Programed death-1, programmed death-ligand 1 (PD-1, PD-L1), and cytotoxic T-lymphocyte antigen-4 (CTLA-4) are immune checkpoint molecules that protect tumor cells from immune attack. T lymphocytes can be restored to their cytotoxic abilities against tumor cells with the use of immune checkpoint inhibitors (ICIs). This treatment, however, is only effective in some patients. As the immunophenotypes of tumor-infiltrating lymphocytes are reported to be correlated with the response rate and prognosis of patients treated with ICIs and ITGAL expression is correlated with the immunophenotypes of tumor-infiltrating lymphocytes, we further investigated whether ITGAL expression correlated with the response and prognosis of patients who underwent ICI therapy. In this dataset, 22 patients had lung adenocarcinoma, 13 patients had lung squamous cell carcinoma, 5 patients had head and neck squamous cell carcinoma, and 25 patients had malignant melanoma. In **Table 5**, the detailed clinical characteristics are shown. The results of the prognostic analysis showed a better outcome for patients with immunotherapy and high ITGAL expression (z=-1.60, P=0.11) (**Figure 6A**). Furthermore, we grouped the patients into responders and non-responders and compared the ITGAL expression between those two groups. Results showed that ITGAL expression was higher in responders than non-responders, although this did not reach statistical significance since the non-responders contained only five cases. (**Figure 6B**).

**Table 5:** Clinical characteristics of immunotherapy datasets.

Characteristics	Tumor (N=65)
Age	
<65	43 (66.15%)
≥65	22 (33.85%)
Gender	
Female	50 (76.92%)
Male	15 (23.08%)
Cancer Type	
LUAD	22 (33.85%)
HEADNECK	5 (7.69%)
MELANOMA	25 (38.46%)
LUSC	13 (20.00%)
Drug	
NIVOLUMAB	28 (43.08%)
PEMBROLIZUMAB	37 (56.92%)

### Immune cell infiltration was linked to anomalous ITGAL expression in NSCLC

TIDE is a method for predicting whether tumors will respond to immunotherapy based on T-cell function. As part of the TIDE algorithm, cytotoxic T-cell analysis (CTL) is used to evaluate the cytotoxicity of T-cells. There was a positive correlation between CTL expression and ITGAL (r=0.561, P=6.77e^-05^) (**Figure 6C**). In order to further explore the relationship between ITGAL and CTL, we investigated the correlation between ITGAL and CTL in the TCGA database and found that ITGAL expression in LUAD and LUSC was positively correlated with CTL levels. (**Figures 6D, E**).

**Figure 7:**
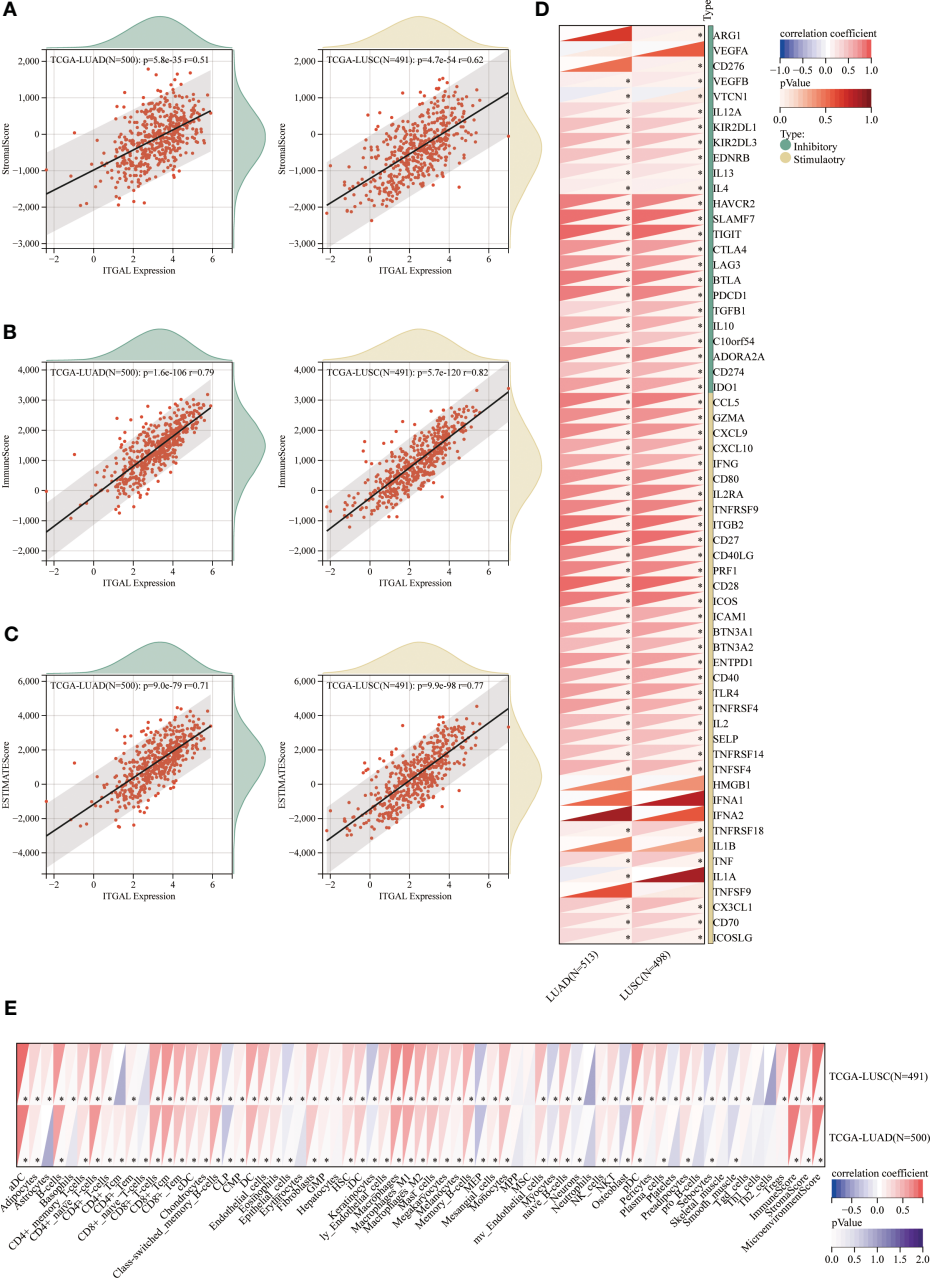
Relationship between immune cell infiltration and aberrant ITGAL expression in NSCLC detected by the TCGA database. **(A-C)** Relationship of ITGAL expression with estimated LUAD and LUSC scores, immunity scores, and stromal scores. **(D)** Relationship between ITGAL and immune checkpoint genes in LUAD and LUSC. **(E)** Differences in immune cell expression between high and low ITGAL groups in LUAD and LUSC. *p<0.05.

Among NSCLC patients, Spearman’s analysis revealed a positive correlation between ITGAL expression and immune scores (estimated score, immune score, and interstitial score). (**Figures 7A–C**). To further elucidate the immune properties of ITGAL related to the tumor immune microenvironment, we evaluated genes involved in immune stimulation and found that 30 genes were positively associated with ITGAL in LUAD and LUSC (**Figure 7D**). According to the TCGA database, CD4+ T cells, CD8+ T cells, and macrophages from patients with NSCLC expressed ITGAL significantly more than CD8+ T cells (**Figure 7E**). ITGAL may be a potential prognostic marker of immunotherapy efficacy for NSCLC when combined with our multicolor immunofluorescence results.

## Discussion

In this study, we performed ITGAL immunohistochemistry and multicolor immunofluorescence on tissue microarrays containing 118 patients with NSCLC to characterize the expression of ITGAL in NSCLC and whether there is a relationship between it and immune cell infiltration. We found that ITGAL was enriched in the mesenchyme in NSCLC tumor tissues, while ITGAL-expressing regions in tumor tissues were accompanied by immune cell aggregation and high expression of immune cell markers. However, ITGAL expression was lower in NSCLC than in normal tissues. This further suggests that ITGAL functions as a protective factor *in vivo* and has the potential to be a biomarker for assessing the immune microenvironment in NSCLC patients.

ITGAL is a cell adhesion molecule that is essential for the migration and adhesion of immune cells in inflammation and immune responses (20, 21). In current oncology studies, expression of the ITGAL gene has been associated with the progression and aggressiveness of renal (22), ovarian and colorectal cancers (23, 24). However, its expression and clinical relevance in NSCLC is unknown. We found that ITGAL expression was significantly down-regulated in NSCLC cells compared to normal lung epithelial cells by including analysis of the TCGA database, the GEO database, and our collection of tissue mRNA from NSCLC patients. Similarly, at the protein level, ITGAL showed different expression patterns under different circumstances. To begin with, we found that tumor tissues expressed less ITGAL protein than healthy tissues. It appears that ITGAL may function as a tumor suppressor in NSCLC. The second finding was that ITGAL expression was significantly higher in the tumor tissues of female patients than in the tumor tissues of male patients, suggesting that the tumor immune microenvironment differs between females and males. Finally, our results showed that the protein expression level of ITGAL was significantly higher in stage I tumors than in stage II or III–IV tumors, indicating that ITGAL was associated with the progression of lung cancer, including disease stage and degree of tumor differentiation.

In the tumor immune microenvironment, immune cell infiltration has been shown to play a role in cancer development (25). The integrin α-L chain encoded by the ITGAL gene is involved in regulating immune cell activity in the tumor microenvironment (7). In T-cell immunotherapy, ITGAL proteins may be involved in tumor recognition by immune cells as the starting point for cytotoxic T cells to generate immune synapses with cancer cells. In recent studies, ITGAL has been found to play a role in the generation of primary resistance to NSCLC immunotherapy (7, 26). In our study, we also found that ITGAL expression correlated not only with the number of multiple immune cells infiltrated but also with the mode of infiltration. Its main site of expression was the tumor stroma, while less expression was found on the surface of tumor cells. Therefore, ITGAL may play a greater role in regulating immune cell function in the tumor microenvironment (27). Gastric cancer has been found to express ITGAL significantly more than peritumor samples in several studies. A high ITGAL expression was significantly associated with the type of sample, the subgroup, the tumor stage, the lymph node stage, and the low survival rate for gastric cancer (12, 19). In contrast, our study found that non-small-cell lung cancer expressed low levels of ITGAL, while controls expressed high levels. Some studies have reported that the upregulation of ITGAL expression was closely associated with the level of infiltration of immunomodulators, chemokines, CD8+, CD4+ T cells, B cells, monocytes, neutrophils, macrophages, T cell modulators, NK cells, and myeloid dendritic cells in gastric adenocarcinoma (STAD) (19). A multicolor immunofluorescence staining and score analysis were used to investigate the relationships between ITGAL and different types of immune cells. Immunofluorescence staining showed that CD4, CD68, CD2, and CD8 non-infiltrating groups had lower infiltration of ITGAL-expressing cells, whereas the highly infiltrating group had higher infiltration of ITGAL-expressing cells. This suggests that ITGAL may play a role in regulating immune cell infiltration, affecting the organism’s tumor microenvironment and immune response, and may play a moderately important role in immune escape, leading to tumor immune alterations that result in a poor prognosis for patients with NSCLC. Further research is needed to determine the exact mechanism by which ITGAL affects immune infiltration in NSCLC.

In conclusion, this study was the first to demonstrate the role of ITGAL in promoting immune cell infiltration in NSCLC by immunohistochemistry and multiplex immunofluorescence and confirmed the important role of ITGAL in the immune microenvironment of NSCLC using public databases. The role of ITGAL and its ligands in the tumor microenvironment has been considered as a potential therapeutic target. Studies have been conducted to explore ways to inhibit the invasion and metastasis of tumor cells by interfering with ITGAL-ICAM interactions (28). It has been shown that the expression level of ITGAL is associated with the efficacy of ICI in patients with NSCLC (29), suggesting that ITGAL might be useful as a molecular marker of NSCLC and as a predictor of ICI efficacy. However, there are several limitations. First, our study only focused on the correlation between ITGAL and immune cell infiltration in NSCLC; the specific mechanism of ITGAL involvement in immune infiltration in NSCLC is unclear and requires further investigation. Second, bioinformatics and patient tissue samples have only been used to validate the relationship between ITGAL and immune cell infiltration. Considering the complexity of the tumor immune microenvironment, animal models may provide a better understanding of ITGAL’s role in NSCLC immunity. The purpose of this study was to examine the relationship between ITGAL expression and immune infiltration and its impact on NSCLC patients’ prognoses. This adds to our understanding of the critical role of ITGAL in human tumors, including NSCLC.

## Conclusions

Our present study provided evidence that increased ITGAL expression promoted immune cell aggregation in NSCLC and may be used as a biomarker for immune infiltration. Furthermore, ITGAL expression may be used to predict the efficacy of ICI therapy in patients with NSCLC treated with ICIs. There are multiple possible roles for ITGAL in regulating immune cell infiltration in NSCLC tissues, as revealed by our study; however, the molecular mechanisms of ITGAL in tumorigenesis and its clinical application prospects warrant further investigation.

## Data availability statement

Publicly available datasets were analyzed in this study. This data can be found here: TCGA: TCGA-LUAD, TCGA-LUSC; GEO: GSE116959, GSE68517, GSE30219, GSE33523, GSE93157.

## Ethics statement

The studies involving humans were approved by Tianjin Medical University Ethics Committee. The studies were conducted in accordance with the local legislation and institutional requirements. The participants provided their written informed consent to participate in this study.

## Author contributions

RZ: Data curation, Investigation, Methodology, Software, Visualization, Writing – original draft, Writing – review & editing. GZ: Data curation, Formal analysis, Software, Validation, Writing – review & editing. ZL: Data curation, Methodology, Software, Writing – review & editing. ZM: Formal analysis, Methodology, Writing – review & editing. HH: Software, Validation, Writing – review & editing. CD: Methodology, Software, Writing – review & editing. YW: Software, Validation, Writing – review & editing. CC: Methodology, Software, Validation, Writing – review & editing. YL: Conceptualization, Data curation, Formal analysis, Funding acquisition, Investigation, Project administration, Resources, Supervision, Writing – review & editing. HL: Conceptualization, Data curation, Formal analysis, Funding acquisition, Investigation, Methodology, Project administration, Resources, Supervision, Writing – review & editing. JC: Conceptualization, Data curation, Formal analysis, Funding acquisition, Investigation, Methodology, Project administration, Resources, Supervision, Validation, Writing – review & editing.
