# Corrigendum: ITGAL expression in non-small-cell lung cancer tissue and its association with immune infiltrates

**DOI:** 10.3389/fimmu.2024.1415814

**Published:** 2024-04-26

**Authors:** Ruihao Zhang, Guangsheng Zhu, Zaishan Li, Zhenzhen Meng, Hua Huang, Chen Ding, Yanan Wang, Chen Chen, Yongwen Li, Hongyu Liu, Jun Chen

**Affiliations:** ^1^ Department of Lung Cancer Surgery, Tianjin Medical University General Hospital, Tianjin, China; ^2^ Department of Cardiothoracic Surgery, Linyi People’s Hospital, Linyi, China; ^3^ Department of Anesthesiology, Linyi People’s Hospital, Linyi, China; ^4^ Tianjin Key Laboratory of Lung Cancer Metastasis and Tumor Microenvironment, Tianjin Lung Cancer Institute, Tianjin Medical University General Hospital, Tianjin, China

**Keywords:** ITGAL, immune microenvironment, NSCLC, biomarker, immune cell

In the published article, there was an error in the affiliation(s)for author Yongwen Li ^3*^. Instead of “Yongwen Li ^3*^”, it should be “Yongwen Li ^4*^”.

Additionally, there was an error in **Figures 1**, **2** as published. There is an error in the typographical order of **Figures 1**, **2**, the **Figures 1**, **2** are in reverse order. The corrected **Figure 1** and its caption ITGAL expression was downregulated in primary NSCLC tissue appear below.

**Figure 1:**
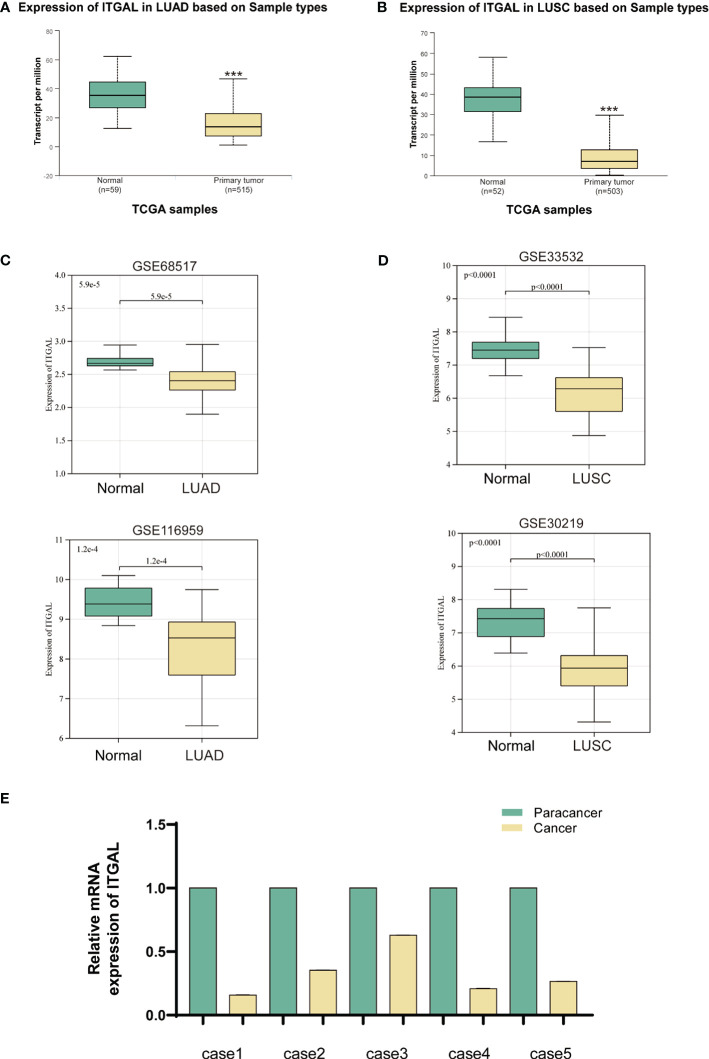


The corrected **Figure 2** and its caption Expression of ITGAL protein and correlation with clinical factors in lung cancer tissue appear below.

**Figure 2:**
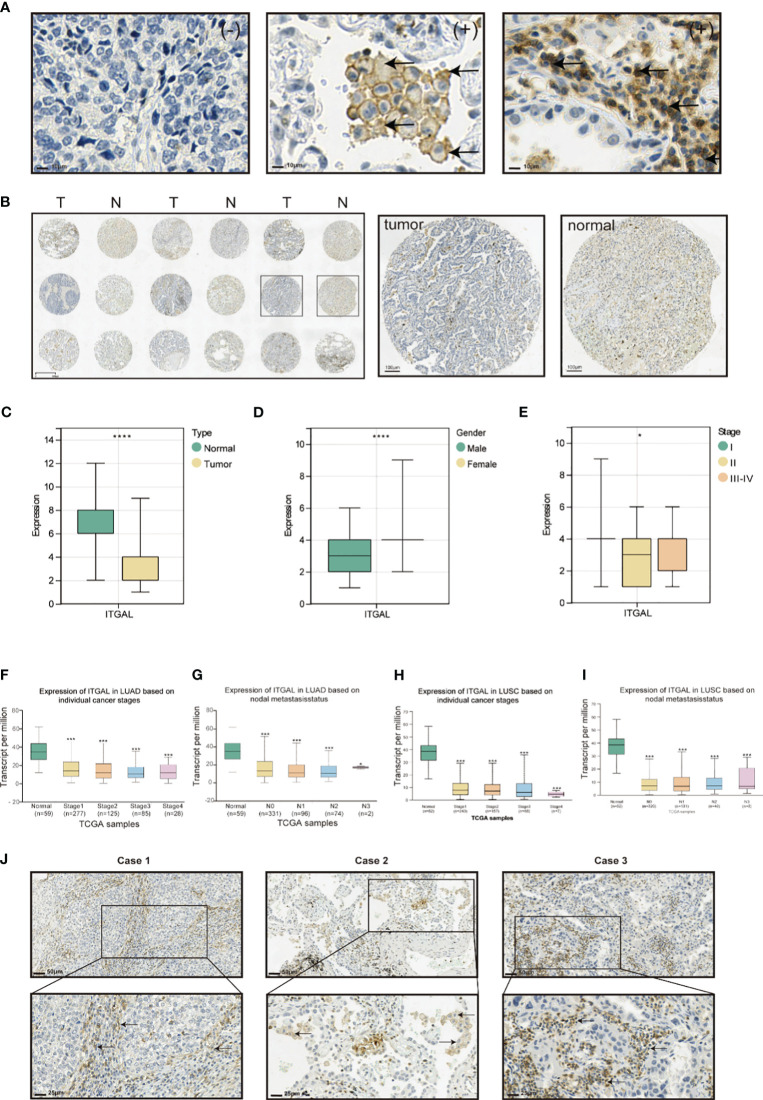


There was also an error in the Funding statement. The funding statement does not clearly state Jun Chen’s funding number: “The author(s) declare that financial support was received for the research, authorship, and/or publication of this article. The National Natural Science Foundation of China grants 82172569 was funded by Yongwen Li. The Tianjin Health Science and Technology Project 5ZC20179 was funded by Hongyu Liu. All other funds were funded by Jun Chen.”

The correct Funding statement appears below.

**FUNDING**

“The author(s) declare that financial support was received for the research, authorship, and/or publication of this article. This study was supported by the National Natural Science Foundation of China 82172569 (supported by Yongwen Li), 82072595 and 61973232 (supported by Jun Chen), Tianjin Medical Key Discipline (Specialty) Construction Project TJYXZDXK-061B (supported by Jun Chen), Tianjin Health Science and Technology Project TJWJ2022XK005 (supported by Jun Chen), 5ZC20179 (supported by Hongyu Liu) and Beijing Science and Technology Innovation Medical Development Fund grant KC2021-JX-0186-57 (funded by Jun Chen). Funding sources were not involved in study design, data collection and analysis, publication decision or manuscript writing.”

Finally, in the published article, there were some errors in the text. In the results section, due to the change in the order of **Figures 1**, **2**, the references to the figures need to be changed accordingly.

In the results section, ITGAL expression was downregulated in primary NSCLC tissue and Expression of ITGAL protein. Correction has been made to references to figures in paragraphs. This sentence previously stated:

“In order to determine whether human ITGAL plays an oncogenic role in NSCLC, the TCGA and GEO databases were used to examine expression levels of ITGAL in LUAD and LUSC. A significant difference was observed between tumor and paraneoplastic lung samples for ITGAL mRNA expression in TCGA-LUAD and TCGA-LUSC(P<0.001) (**Figures 2A, B**). For further validation, we detected the expression of ITGAL in LUAD by GEO databases, GSE68517 and GSE116959, and in LUSC by databases GSE33532 and GSE30219. A significant reduction in ITGAL protein levels was observed in LUAD and LUSC compared to normal tissues (**Figures 2C, D**). In a subsequent study, we examined the expression of ITGAL mRNA in tumor and peritumor lung tissue samples from five patients with NSCLC. Tissues from tumors expressed less ITGAL than healthy tissues (**Figure 2E**).”

The corrected sentence appears below:

“In order to determine whether human ITGAL plays an oncogenic role in NSCLC, the TCGA and GEO databases were used to examine expression levels of ITGAL in LUAD and LUSC. A significant difference was observed between tumor and paraneoplastic lung samples for ITGAL mRNA expression in TCGA-LUAD and TCGA-LUSC(P<0.001) (**Figures 1A, B**). For further validation, we detected the expression of ITGAL in LUAD by GEO databases, GSE68517 and GSE116959, and in LUSC by databases GSE33532 and GSE30219. A significant reduction in ITGAL protein levels was observed in LUAD and LUSC compared to normal tissues (**Figures 1C, D**). In a subsequent study, we examined the expression of ITGAL mRNA in tumor and peritumor lung tissue samples from five patients with NSCLC. Tissues from tumors expressed less ITGAL than healthy tissues (**Figure 1E**).”

In the results section, Correlation with clinical factors in lung cancer tissue. Correction has been made to references to figures in paragraphs. This sentence previously stated:

Tissue microarrays containing tissue from 118 patients with primary NSCLC and paired paracancerous tissues were used for ITGAL IHC staining and scoring. As shown in the table below, the tissue microarrays contained characteristics about patients with lung cancer (Table 1). The IHC results showed that ITGAL was mainly distributed in the plasma membrane and the cell membrane, with a patchy or nested distribution. In accordance with the median score of ITGAL, patients were divided into two groups: those with high ITGAL expression and those with low ITGAL expression (**Figure 1A**). The following table presents the results of the ITGAL analysis stratified by the median immunohistochemical score into high and low expression for each clinical factor (Table 2). By analyzing the IHC results of each patient, we found that paraneoplastic tissues had significantly higher ITGAL expression compared to matched tumor tissues (**Figures 1B, C**). The expression of ITGAL was higher in tumor tissues from female patients than male patients, and in tumor tissues from early-stage patients than advanced-stage patients (**Figures 1D, E**). In order to further validate our findings, we examined ITGAL expression at various stages of NSCLC tumor progression and lymph node metastases using the TCGA database. The ITGAL expression of LUAD and LUSC at stratified clinical stages were lower than that of paracancer normal tissues (**Figures 1F, H**). Meantime, the ITGAL expression of LUAD and LUSC at stratified N stages were lower than that of paracancer normal tissues (**Figures 1G, I**).

Moreover, ITGAL was prominently highly expressed in the stroma area of the tumor tissues and on the membrane and cytoplasm of macrophages and lymphocytes aggregated in these areas (**Figure 1J**).

The corrected sentence appears below:

Tissue microarrays containing tissue from 118 patients with primary NSCLC and paired paracancerous tissues were used for ITGAL IHC staining and scoring. As shown in the table below, the tissue microarrays contained characteristics about patients with lung cancer (Table 1). The IHC results showed that ITGAL was mainly distributed in the plasma membrane and the cell membrane, with a patchy or nested distribution. In accordance with the median score of ITGAL, patients were divided into two groups: those with high ITGAL expression and those with low ITGAL expression (**Figure 2A**). The following table presents the results of the ITGAL analysis stratified by the median immunohistochemical score into high and low expression for each clinical factor (Table 2). By analyzing the IHC results of each patient, we found that paraneoplastic tissues had significantly higher ITGAL expression compared to matched tumor tissues (**Figures 2B, C**). The expression of ITGAL was higher in tumor tissues from female patients than male patients, and in tumor tissues from early-stage patients than advanced-stage patients (**Figures 2D, E**). In order to further validate our findings, we examined ITGAL expression at various stages of NSCLC tumor progression and lymph node metastases using the TCGA database. The ITGAL expression of LUAD and LUSC at stratified clinical stages were lower than that of paracancer normal tissues (**Figures 2F, H**). Meantime, the ITGAL expression of LUAD and LUSC at stratified N stages were lower than that of paracancer normal tissues (**Figures 2G, I**).

Moreover, ITGAL was prominently highly expressed in the stroma area of the tumor tissues and on the membrane and cytoplasm of macrophages and lymphocytes aggregated in these areas (**Figure 2J**).

The authors apologize for these errors and state that they do not change the scientific conclusions of the article in any way. The original article has been updated.

